# The combination of lactoferrin and linolenic acid inhibits colorectal tumor growth through activating AMPK/JNK-related apoptosis pathway

**DOI:** 10.7717/peerj.11072

**Published:** 2021-05-31

**Authors:** Qianqian Yao, Huiying Li, Linlin Fan, Shengnan Huang, Jiaqi Wang, Nan Zheng

**Affiliations:** 1 Key Laboratory of Quality & Safety Control for Milk and Dairy Products of Ministry of Agriculture and Rural Affairs, Institute of Animal Sciences, Chinese Academy of Agricultural Sciences, Beijing, China; 2 Laboratory of Quality and Safety Risk Assessment for Dairy Products of Ministry of Agriculture and Rural Affairs, Institute of Animal Sciences, Chinese Academy of Agricultural Sciences, Beijing, China; 3 State Key Laboratory of Animal Nutrition, Institute of Animal Sciences, Chinese Academy of Agricultural Sciences, Beijing, China

**Keywords:** Lactoferrin, Oleic acid, DHA, Linolenic acid, HT29 cells

## Abstract

Colorectal cancer is a common cause of death with few available therapeutic strategies, and the preventative complexes in adjunctive therapy are urgently needed. Increasing evidences have shown that natural ingredients, including lactoferrin, oleic acid, docosahexaenoic acid (DHA) and linolenic acid, possess anti-inflammatory and anti-tumor activities. However, investigations and comparisons of their combinations in colorectal tumor model have not been reported, and the mechanism is still unrevealed. In the study, we examined the viability, migration, invasion and apoptosis of HT29 cells to choose the proper doses of these components and to select the effective combination in vitro. BALB/c nude mice bearing colorectal tumor were used to explore the role of selected combination in inhibiting tumor development in vivo. Additionally, metabonomic detection was performed to screen out the specific changed metabolitesand related pathway. The results demonstrated that lactoferrin at 6.25 μM, oleic acid at 0.18 mM, DHA at 0.18 mM, and linolenic acid at 0.15 mM significantly inhibited the viabilities of HT29 cells (*p* < 0.05). The combination of lactoferrin (6.25 μM) + linolenic acid (0.15 mM) exhibited the strongest activity in inhibiting the migration and invasion of HT29 cells in vivo and suppressing tumor development in vitro (*p* < 0.05). Furthermore, the lactoferrin + linolenic acid combination activated p-AMPK and p-JNK, thereby inducing apoptosis of HT29 cells (*p* < 0.05). The present study was the first to show that lactoferrin + linolenic acid combination inhibited HT29 tumor formation by activating AMPK/JNK related pathway.

## Introduction

As one of the most prevalent types of cancer worldwide, colorectal cancer (CRC) is associated with high mortality (Bray et al., 2018). In China, CRC was one of the most common cancers in 2015, with 225,000 male and 162,600 female cases (Wu et al., 2020). Despite a decrease in morbidity and increase in survival rates due to improvements in preventive programs, screening strategies, and chemotherapy methods, the five-year survival rate of CRC is about 50–60%, even lower in several countries, such as Slovakia, Poland, Croatia, etc. (Allemani et al., 2018). Currently, the most common treatments for the disease are surgery, radiation therapy and chemotherapy accompanied by tremendous pain and huge costs for health care. Therefore, a good understanding of proper strategies preventing CRC will benefit the human welfare. Statistical data indicate that the majority of CRC cases are associated with the environment, such as lifestyle and diet (Hullings et al., 2020; Byrd et al., 2020). In return, dietary interventions, especially natural food ingredients, combined with adjunctive therapy, may play an important role in preventing and inhibiting CRC.

Accumulating evidences show that several polyunsaturated fatty acids (PUFAs), mainly linolenic acid (LA), docosahexaenoic acid (DHA) and oleic acid (OA), in foods make a contribution for the prevention and inhibition of various tumor growth. A meta-analysis study from Psaltopoulou et al. (2011) found that a higher olive oil consumption made a significant 30% lower probability of gastrointestinal cancers, and this property is mainly attributed to several monounsaturated fats, especially OA. Song et al. (2011) showed that DHA exerted activity to suppress PANC-1 and SW1990 cells (pancreatic cancer cell line) growth through downregulating the WNT/β-catenin reacted signaling. The anti-tumor bioactivity of LA was demonstrated by Llor et al. (2003) who found that LA induced apoptosis of colorectal cancer cells line (HT29 cells). Researches exploring the anti-tumor mechanism of PUFAs have demonstrated that they may exert stronger anti-tumor activity by generating complexes combining with bioproteins than PUFAs or proteins alone. HAMLET (human alpha-lactalbumin made lethal to tumor cells) is a widely acknowledged complex formed by α-lactalbumin and OA, and could selectively kill cancer cells (Svensson et al., 2000). OA and LA could induce the formation of amorphous aggregates of the intermediate of α-lactalbumin in a concentration-dependent and time-dependent manner, and then induced apoptosis in cancer cells (Zhang et al., 2009). Xing, Liu & Hao (2016a, 2016b*)* found that the ovalbumin-OA complex, similar with the HAMLET, significantly suppressed Caco-2 cells growth, blocked Caco-2 cells cycle and induced Caco-2 cells apoptosis, when compared with OA treatment alone. Fang et al. (2015) showed that β-lactoglobulin-OA and β-lactoglobulin-LA complexes had ability to decrease the cell viability and induce apoptosis of MCF-7 and HeLa cells, however, β-lactoglobulin, OA and LA alone did not exert anti-tumor activity.

Lactoferrin (80 kDa, LF), a greatly recognized multifunctional protein, is a single polypeptide chain glycoprotein with two lobes, and can be found in various body fluids, especially in colostrum (Małaczewska & Rotkiewicz, 2007). The biological properties of LF, such as anti-bacterial, anti-viral, anti-tumor, anti-inflammatory and anti-carcinogenic, have been widely reported (Li et al., 2019; Koshu et al., 2016). LF is an effective regulator of innate immunity and plays an important role in improving host defense system. It can not only defend microbial infections through recognizing the LF-binding protein (Kouichirou et al., 2005; Aguilera et al., 1998), but also prevent inflammatory diseases, such as allergies, arthritis and acute lung injury (Hayashida et al., 2004). More importantly, LF has been reported to inhibit and resist many types of cancers, including gastric cancer (Xu et al., 2010), breast cancer (Wang et al., 2012), renal cancer (Lewis & Hayes, 2011) and CRC (Li et al., 2017), indicating that LF is a potential anti-cancer protein.

Given that the anti-tumor activity of exogenous PUFAs and specific bioprotein could be elevated by the interaction between them, we speculated that the ability of lactoferrin (LF) to inhibit cancer cells growth also could be enhanced by combining with PUFAs. However, the combination of LF and PUFAs is rare to study and the combination effect on cancer cells remains unrevealed. In the present study, we investigated and compared the anti-tumor effects of LF and OA/DHA/LA combinations in a colorectal tumor model. And the AMPK/JNK related pathway was investigated to reveal the underlying mechanisms. Combining with further pharmacokinetics studies, the results might provide a novel dietary intervention for colon cancer.

## Materials and Methods

### Chemicals

Bovine LF (>85% purity, Cat#L9507), LA (C18:3, cis,cis,cis-9,12,15, ≥99% purity, Cat#L2376), DHA (C22:6 *n−*3, ≥98% purity, Cat#D2534), OA (C18:1, ≥99% purity, Cat#O1383) and AMPK inhibitor dorsomorphin (Compound C, Cat#171261) were purchased from Sigma (St. Louis, MO, USA). 5-fluorouracil (5-Fu, ≥98% purity, Cat#F8300) was obtained from Solarbio (Beijing, China). LF and 5-Fu was diluted with PBS to 10 mg/mL stocking solution, and DHA/LA/OA was diluted with absolute ethyl alcohol to 10 mg/mL stocking solution, respectively. Primary antibodies against β-actin (Cat#sc-47778), AMPK (Cat#sc-74461), JNK (Cat#sc-7345), p-JNK (Cat#sc-6254), caspase-3 (Cat#sc-56053), Bax (Cat#sc-7480), Bcl-2 (Cat#sc-7382) were purchased from Santa Cruz Biotechnology (Santa Cruz, CA, USA), and Ap-AMPK (Cat#PA5-17831) was obtained from Invitrogen (CA, USA). Anti-cleaved Caspase-3 (Cat# ab49822) was purchased from Abcam (Cambridge, UK).

HT29 (human colon cancer cell line, Cat#CL-0118), DLD1 (human colon cancer cell line, Cat#CL-0074), HCT116 (human colon cancer cell line, Cat#CL-0096), AGS (human gastric epithelial cell line, Cat#CL-0022) and HepG2 (human hepatoma cell line, Cat#CL-0103) were provided by Procell Life Science & Technology Co., Ltd. (Wuhan, China).

### Cell culture and viability detection

HT29 and AGS cells were cultured in DMEM medium with 10% FBS. DLD1, HCT116 and HepG2 cells were cultured in RPMI-1640 medium supplemented with 10% FBS. All of the different cells were cultured at 37 °C in 5% CO_2_ and 95% saturated atmospheric humidity.

The HT29 cells were seeded onto a 96-well plate (2 × 10^4^ cells/well) and cultivated in 100 μL medium for 24 h, then FBS-free medium supplemented with different concentrations of LF, OA, DHA, LA were used to replace the old ones and cultivated for 48 h, and 5-Fu was used as positive control. Then the CCK-8 kit was utilized accordance with the instructions. The detection of cell survival rate and viability data analysis were performed according to Li et al. (2019), and the IC_50_ of four chemicals were calculated. The dosages resulting in viability >90% with statistical significance were selected as the final concentrations of LF and fatty acids.

We further explore the inhibitory effect of four chemicals at the selected concentration and IC_50_ on other human cancer lines (DLD1, HCT116, HepG2 and AGS). The cells were seeded onto a 96-well plate for 24 h, then FBS-free medium with LF, OA, DHA, LA were used to replace the old ones and cultivated for 48 h, and 5-Fu was used as positive control. Then the CCK-8 kit was utilized accordance with the instructions.

### Cell apoptosis detection

To evaluate the apoptotic death of HT29 cells under different treatments, the apoptosis detection was conducted (Hsieh et al., 2012). Briefly, cells were cultured in 6-well plates and treated with LF (6.25 μM), OA (0.18 mM), DHA (0.18 mM), LA (0.15 mM), 5-Fu (2.0 μM), LF + OA, LF + DHA, or LF + LA for 48 h. According to the protocol of the Annexin V-FITC/PI apoptosis detection kit (Beyotime, Shanghai, China), the cells were resuspended in binding buffer (250 μL) after washed by PBS buffer. Annexin V-FITC and PI buffer(10, 20 g/L respectively)were added to the cell suspension. Cell samples should be slightly vortexed and incubated for 10 min in the dark (25 °C), and then add 300 μL binding buffer to each tube. Samples were detected by flow cytometry within 2 h. Cells stained AV+/PI+ (right upper quadrant) were considered as necrotic and late apoptotic, AV+/PI− (right lower quadrant) as early apoptotic, and AV−/PI− (left lower quadrant) as viable ones.

### Cell invasion detection

The cell invasion detection was conducted as described previously (Orucevic et al., 1999). Transwell chambers (Corning^®^ BioCoat^™^ Matrigel^®^, USA) were used in the cell invasion detection method. The experiment was designed according to the following principle, which demonstrated that cancer cells (as HT29 cells) tended to migrate into the outer chambers containing 10% FBS. HT29 cells were cultured (1 × 10^4^ cells/well) in 150 μL serum-free medium, and LF (6.25 μM), OA (0.18 mM), DHA (0.18 mM), LA (0.15 mM), 5-Fu (2.0 μM), LF + OA, LF + DHA, or LF + LA was added to the insert respectively. Meanwhile 500 μL RPMI-1640 complete medium (supplemented with 10% FBS) were added in the outer chambers. Cells were cultured for 24 h, and cotton swabs were used to scrub the filters surfaces. Migrated cells were treated with ice-cold methanol for 15 min, while the undersides were dyed by 0.2% crystal followed by washing with ice-cold PBS (3 min × 5). The dyed cells on the undersurfaces were counted and calculated in three random fields.

### Cell scratch analysis

To evaluate the migration ability of HT29 cells under different treatments, we performed the cell scratch assay as described previously (Shameem et al., 2015). In 6-well plates, HT29 cells were cultivated until the density was higher than 85%. A mechanical scratch across the cells with about 2.5 mm width was made, and then cells were treated with LF (6.25 μM), OA (0.18 mM), DHA (0.18 mM), LA (0.15 mM), 5-Fu (2.0 μM), LF + OA, LF + DHA, or LF + LA, respectively. The width of cells was photographed and analyzed at 0 h and 24 h later. The capacity of LF, unsaturated fatty acids, or combinations to suppress migration of HT29 cells were reflected by the changes of scratch width (0 h vs 24 h). The recovery rate (%) was calculated as the changes of scratch width in the treatment groups within 24 h/the ones in control group at 0 h ×100%.

### Animal model construction

Sixteen male BALB/c nude mice (18–22 g) were obtained from Beijing Vital River Laboratory Animal Technology Co., Ltd. (Beijing, China). The animal experiments were approved by the Ethics Committee of the Chinese Academy of Agriculture Sciences (Beijing, China) (Permission number: CAS20190410, 10th April, 2019), conforming to internationally accepted principles in the care and use of experimental animals (National Research Council (NRC), 2011). Animals were kept in cages at a constant temperature of 25 °C and relative humidity of 55%, with 12 h light/dark cycle. The mice had free access to standard rodent chow and water ad libitum throughout the study duration except during actual measurements and were acclimatized for at least 7 days.

HT29 cells (5 × 10^7^ cells per dish) in 200 μL Matrigel medium (BD) were subcutaneously injected into the left flank of each nude mouse. When the tumor volume reaches 90–110 mm^3^, the nude mice were randomly assigned into four groups (*n* = 4) as follows: control (without treatment), LF group (50 mg/kg b.w.), LA group (5 mg/kg b.w.), and LF + LA group. The mice were administered LF, LA, or combinations orally daily at the same time. All mice were euthanized on the 25th day using sodium pentobarbital anesthesia (50 mg/kg body weight), and all efforts were made to minimize the suffering of the mice, then the tumors were weighed. Tumor diameters were recorded with a caliper every 4 days, and the relative tumor volume (RTV, %) and relative tumor proliferation rate (%) were calculated according to Li et al. (2017).

### Metabonomics detection of HT29 cells

Significantly changed metabolites in HT29 cells treated with LF (6.25 μM), LA (0.15 mM), or (LF + LA) were measured. The cell samples (10^6^ per well) were gathered and washed with ice PBS buffer twice, and then the cells were lysed by the lysis solution (70% methanol + 0.1% formic acid). After vortex for 10 s and ultrasonication for 20 min, the lysate was centrifuged at 10,000×*g* for 10 min at 4 °C, and the supernatant was collected. Metabolites in HT29 cells were performed by a UHPLC system (Ultimate 3000; Dionex, Sunnyvale, CA, USA) equipped with a Waters Column (Acquity BEH C18 1.7 µm, 2.1 × 50 mm) at 40 °C. The following detection steps were cited in reference (Li et al., 2018).

### Western blotting detection

The western blotting was performed as described previously in Li et al. (2017). A total of 10 mg tumor tissue or 10^6^ HT29 cells were treated with 0.75 mL RIPA buffer, and homogenized using a Sonifier (3 × 5 min) and then centrifuged (8,000*g* × 5 min). All the operations were performance at 4 °C. The supernatant was collected and measured using the BCA kit. After heating for 10 min, the protein samples were loaded and separated by SDS-PAGE.

### Statistical analysis

Data were expressed as the mean ± standard deviation (SD) from several independent experiments (*n* ≥ 3). Statistical analyses were performed using SPSS 13.0 (SPSS Inc., USA). Analysis of variance and independent samples *t*-test were used to determine the differences among the treatments. A *p* value <0.05 (*p* < 0.05) was considered statistically significant.

## Results

### Dosage selections of lactoferrin and three unsaturated fatty acids

**Figure 1:**
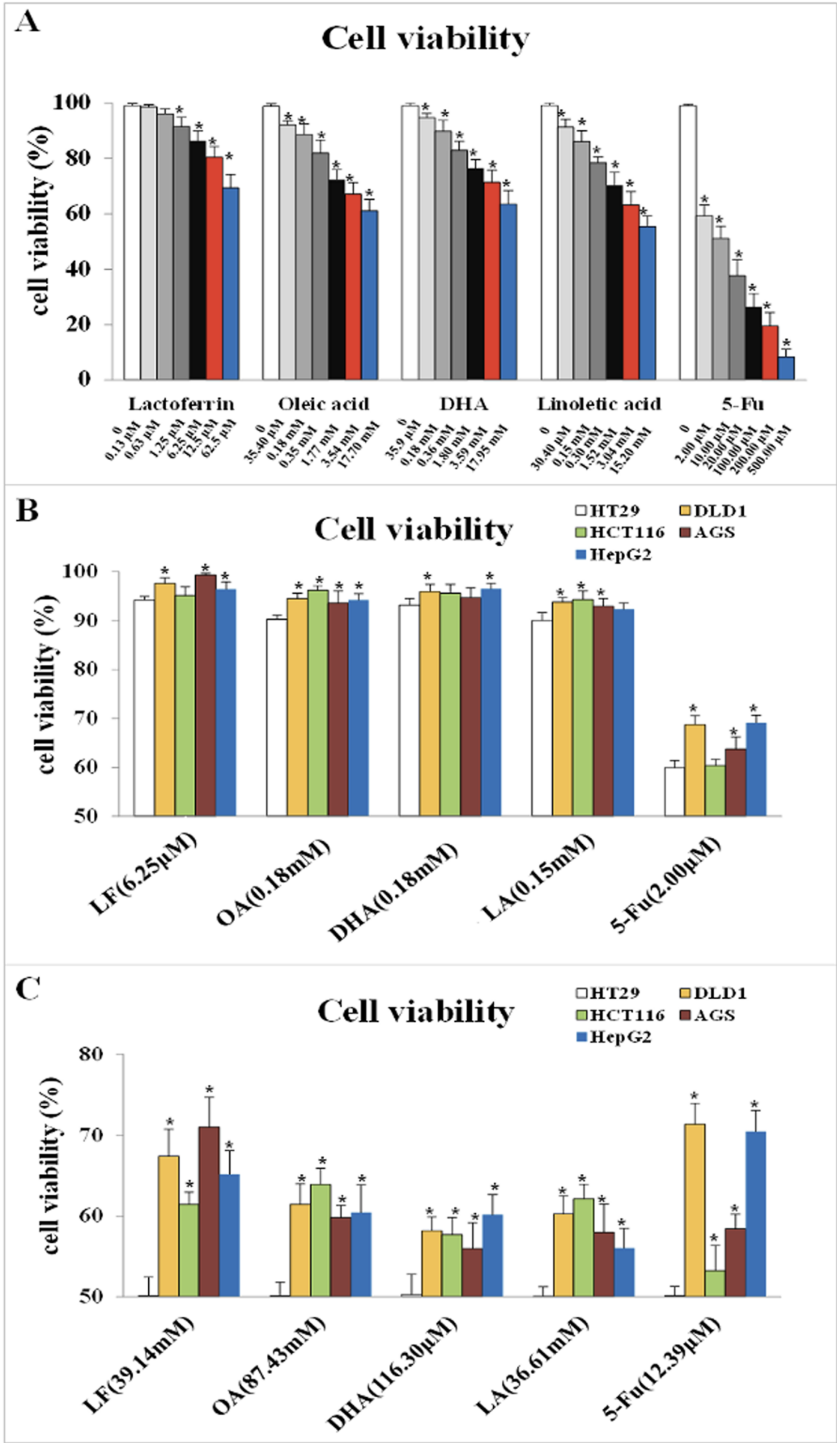
Cell viability treated with lactoferrin and three unsaturated fatty acids and detected by CCK-8 kits. (A) With 5-Fu as the positive control, lactoferrin and three unsaturated fatty acids showed different inhibition on HT29 cells survival rate under 48 h treatment. (B) Comparison the inhibitory effects of lactoferrin (6.25 μM), OA (0.18 mM), DHA (0.18 mM) and LA (0.15 mM) at the chosen concentration in (A) on other four different cancer cell lines. 5-Fu (2.0 μM) was used as the positive control. (C) Comparison the inhibitory effects of lactoferrin (39.14 mM), OA (87.43 mM), DHA (116.30 mM) and LA (36.61 mM) at the IC_50_ concentration (for HT29 cells) on other four different cancer cell lines. 5-Fu was used as the positive control. Significant difference from control was observed by variance analysis and *t*-test comparison, and the data was represented as mean ± SD, *n* = 8. *p < 0.05, compared with the control.

CCK-8 detection was performed to observe the effect of LF and three unsaturated fatty acids on the viability of HT29 cells and to choose their proper dosage. As Fig. 1A showed, LF at 6.25 μM, OA at 0.18 mM, DHA at 0.18 mM, and LA at 0.15 mM inhibited the viability of HT29 cells significantly compared with the control (*p* < 0.05). The IC_50_ values of LF, OA, DHA and LA was 39.14 mM, 87.43 mM, 116.30 μM and 36.61 mM, respectively.

As Fig. 1B showed, at the selected concentration, the inhibitory effects of the four chemicals on HT29 cells growth were higher than other four cell lines, indicating that HT29 cells was more sensitive to LF and the three PUFAs, comparing to other cancer cell lines. Moreover, at the condition of IC_50_ concentration, lactoferrin and three unsaturated fatty acids could inhibit about 50% HT29 cells growth, however, only inhibit 30–40% other cancer cells growth (Fig. 1C).

However, referring to the principles of dosage selection, mainly effectiveness and safety, effectiveness means the cell viabilities with the treatments of the chemicals are significantly lower than the control without any treatment (*p* < 0.05), and safety means the cell viability with the treatments were above 90%. Thus, 6.25 μM of LF and 0.18 mM, 0.18 mM, 0.15 mM of OA, DHA, LA was selected as the proper dosage in subsequent experiments.

### Lactoferrin and three unsaturated fatty acids induced apoptosis of HT29 cells

**Figure 2:**
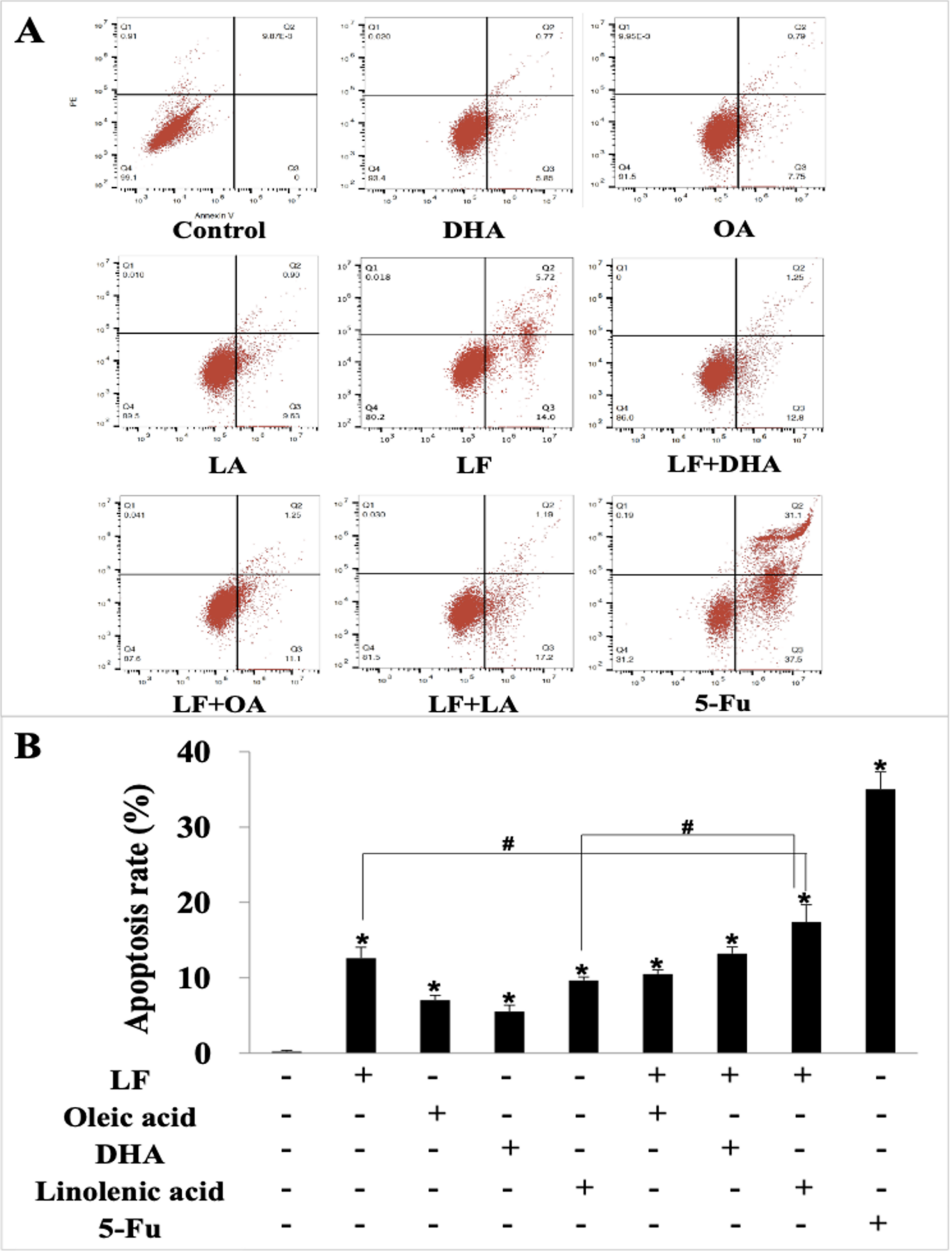
HT29 cell apoptosis treated with lactoferrin and three unsaturated fatty acids and detected by Annexin V-FITC/PI staining. Lactoferrin (6.25 μM), OA (0.18 mM), DHA (0.18 mM) and LA (0.15 mM) and the combinations induced HT29 cells apoptosis in different degrees. 5-Fu (2.0 μM) was used as the positive control. (A) Partitions of HT29 cells. (B) Statistical analysis of cells apoptosis rate (LR region, lower right quadrant). Significant difference from control was observed by variance analysis and *t*-test comparison, and the data was represented as mean ± SD, *n* = 3. *p < 0.05, compared with the control (without any treatment); #*p* < 0.05, compared with the LF + linolenic acid (LA) combination group.

We further explored the effect of LF and three unsaturated fatty acids on apoptosis in HT29 cells by Annexin V-FITC/PI staining. According to the analysis procedures of apoptosis kit, cells in LR region (lower right quadrant) are the early apoptotic ones, thus, we compared LR rate among these groups and found that LF, three unsaturated fatty acids, and the combinations induced apoptosis of HT29 cells to different degrees (*p* < 0.05, when compared the control), and the apoptosis rate was the highest one in the LF + LA group (17.40% ± 2.31%) among all these groups; there were significant differences of the apoptosis rate in LF + LA combination group, when compared with single LF group (12.60% ± 1.45%) or single LA group (9.64% ± 0.46%) (*p* < 0.05, Fig. 2).

### Lactoferrin and three unsaturated fatty acids inhibited invasion and migration of HT29 cells

**Figure 3:**
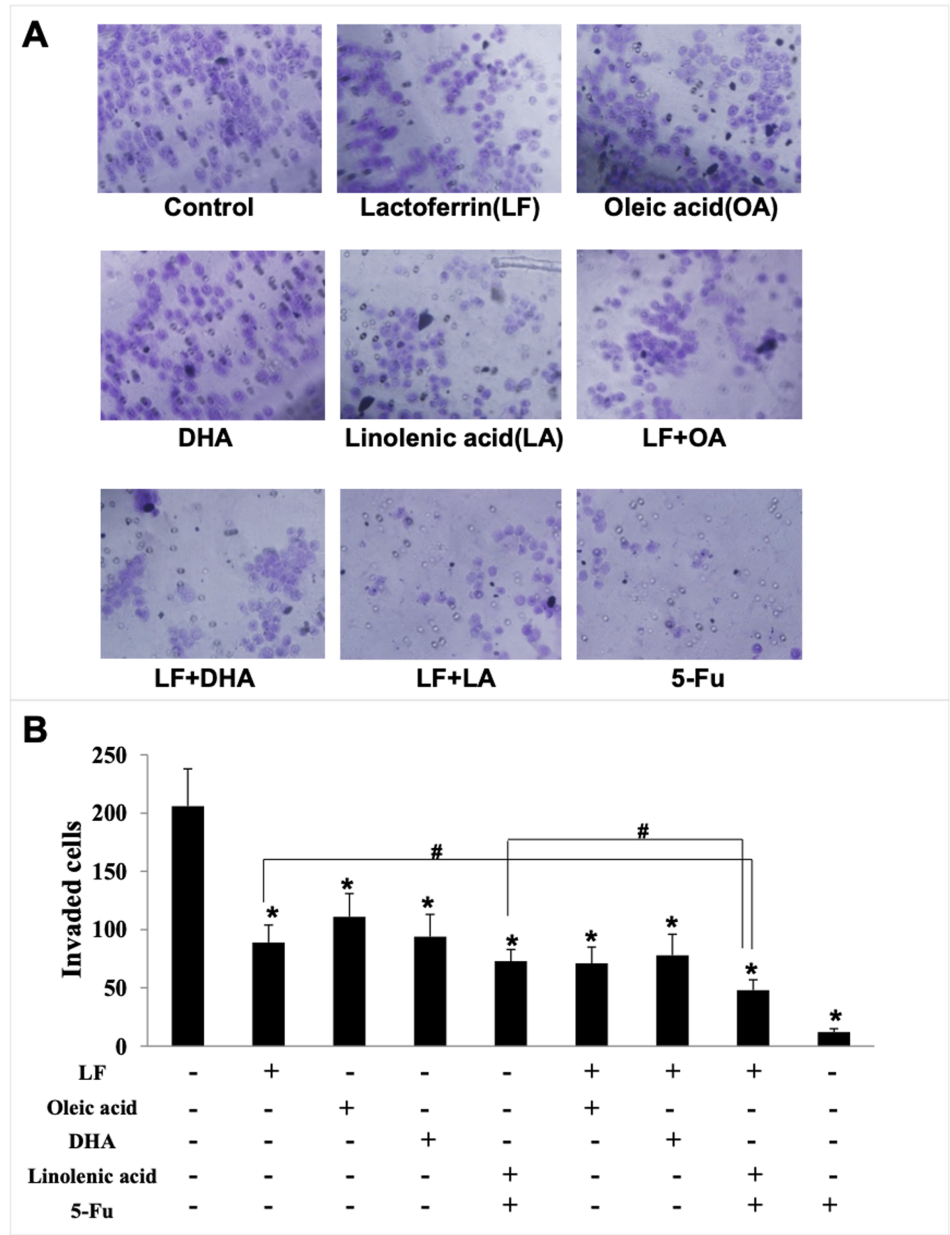
HT29 cell invasion treated with lactoferrin and three unsaturated fatty acids and detected by transwell. Lactoferrin (6.25 μM), OA (0.18 mM), DHA (0.18 mM) and LA (0.15 mM) and the combinations inhibited HT29 cells invasion in different degrees. 5-Fu (2.0 μM) was used as the positive control. (A) Invaded cells stained by crystal violet. (B) Statistical analysis of the invaded cells. Significant difference from control was observed by variance analysis and *t*-test comparison, and the data was represented as mean ± SD, *n* = 3. *p < 0.05, compared with the control. #*p* < 0.05, compared with the LF + LA combination group. 200× magnification.

**Figure 4:**
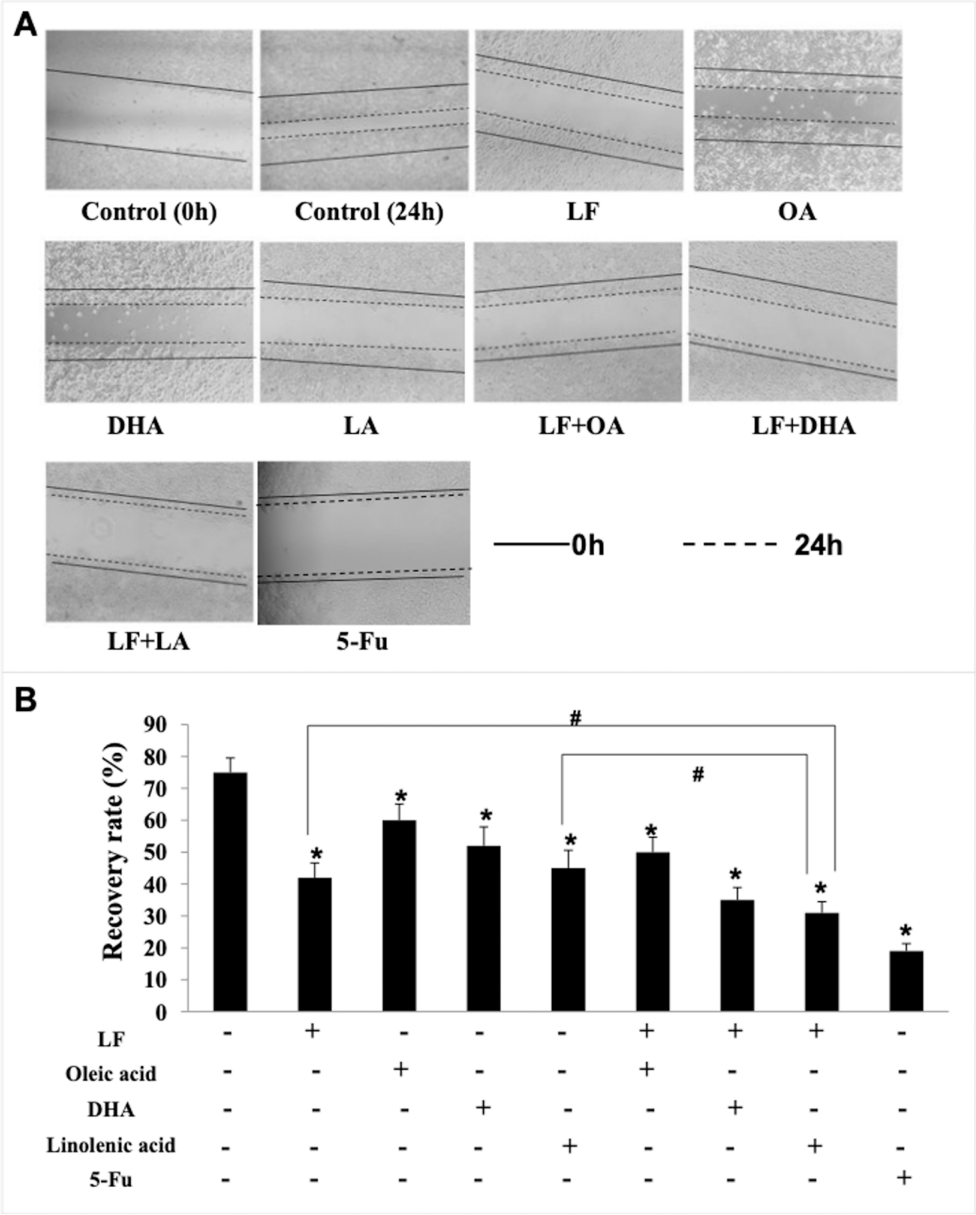
HT29 cell migration treated with lactoferrin and three unsaturated fatty acids and detected by scratch analysis. Lactoferrin (6.25 μM), OA (0.18 mM), DHA (0.18 mM) and LA (0.15 mM) and the combinations inhibited HT29 cells migration in different degrees. 5-Fu (2.0 μM) was used as the positive control. (A) Cells migration photographs. (B) Significant difference from control was observed by variance analysis and *t*-test comparison, and the data was represented as mean ± SD, *n* = 3. *p < 0.05, compared with the control. #*p* < 0.05, compared with the LF + LA combination group. 40× magnification.

Giving that cancer cells have strong ability to migrate and invade into surrounding tissues and then to the target organs (Zhao et al., 2013), the migration/invasion assays were conducted to evaluate the effects of treatments on metastatic ability of HT29 cells. In the transwell assay, the invaded cells on the filters were counted and calculated, and the results demonstrated that when compared with the control (207 ± 32 invaded cells), LF (89 ± 15 invaded cells, *p* < 0.05), OA (111 ± 20 invaded cells, *p* < 0.05), DHA (94 ± 20 invaded cells, *p* < 0.05) and LA (73 ± 10 invaded cells, *p* < 0.05) significantly inhibited cell invasion (Fig. 3). Especially, the lowest number of invaded cells was observed in the LF + LA group (48 ± 9), among all the groups (71 ± 14 invaded cells in LF + OA, 78 ± 17 invaded cells in LF + DHA). In the scratch analysis, it was found that the recovery rate in LF, OA, DHA, LA groups was (42.0 ± 4.36)%, (59.67 ± 5.03)%, (52.0 ± 6.0)%, (45.0 ± 5.29)%, respectively (all *p* < 0.05, compared with the control (75.0% ± 4.58%)), and the lowest rates were observed in the LF + LA group (31.3% ± 3.52%) (Fig. 4).

### Lactoferrin and linolenic acid inhibited tumor growth in a tumor bearing nude mouse model

**Figure 5:**
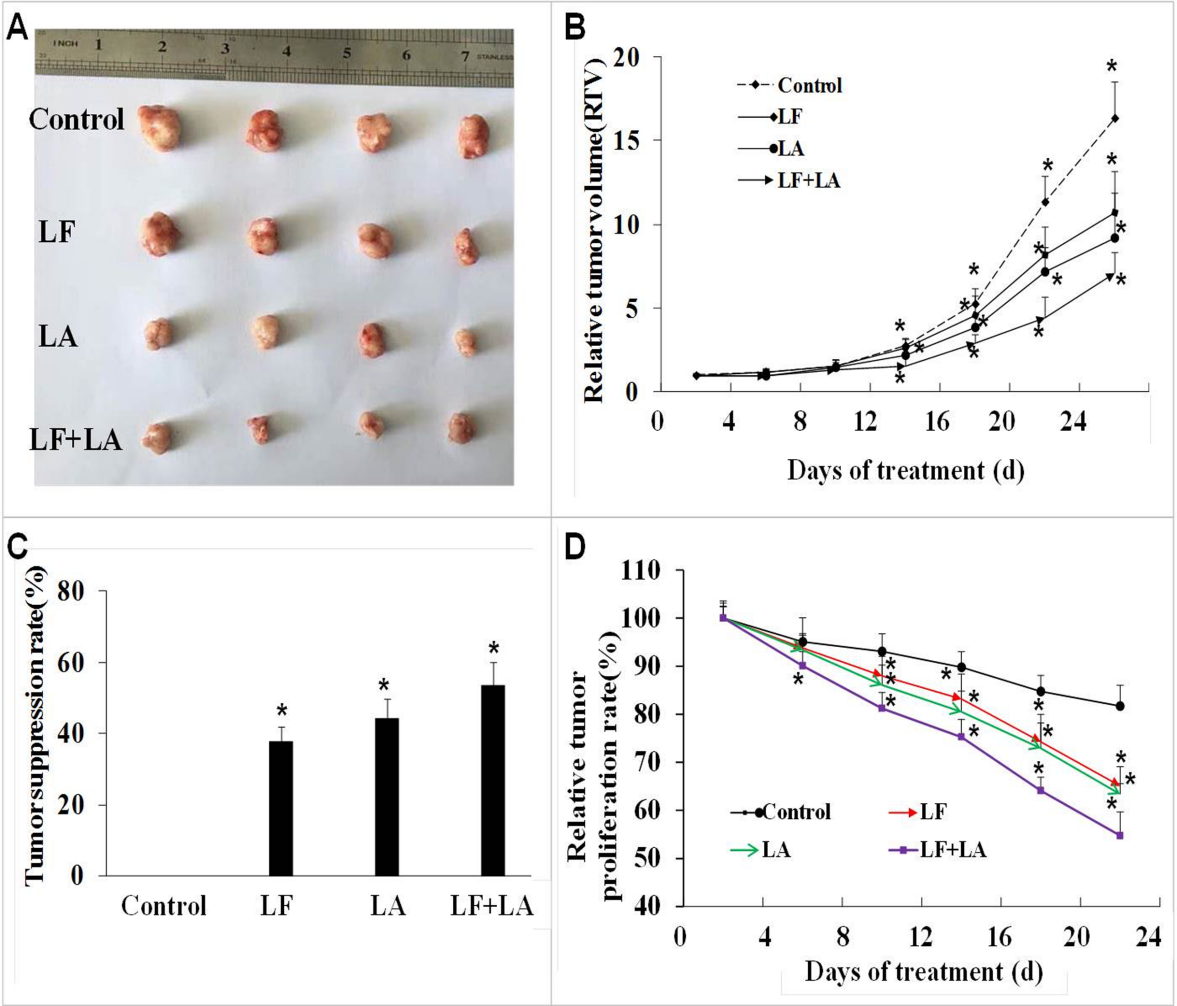
In vivo effect of lactoferrin and linolenic acid on HT29 tumor-bearing nude mice, through oral administration. (A) Treatment of LF (50 mg/kg b.w.) and LA (5 mg/kg b.w.) decreased the size of HT29 tumors. (B) Relative tumor volume, which was calculated by each tumor volume. *p < 0.05, comparing with the control (no treatment), *n* = 4. (C) Tumor suppression rate, which was calculated by each tumor weight. *p < 0.05, comparing with the control (no treatment), *n* = 4. (D) Relative tumor proliferation rate, which was calculated by relative tumor volumes of different groups. *p < 0.05, comparing with the control (no treatment), *n* = 4.

Based on the in vitro results, we selected the LF + LA for in vivo experiments. The BALB/c nude mouse xenograft model was established, and these two compounds alone or its combination were delivered into the mice through oral administration. On day 25th, the RTV and the relative tumor proliferation rates in LF + LA group was 7.17% ± 1.18% and 54.77% ± 4.93%, which were much lower than the ones in control group (16.37% ± 2.18%, 81.71% ± 4.33%), LF group (10.73% ± 2.43%, 65.08% ± 4.01%), and LA group (9.26% ± 2.65%, 63.37% ± 2.16%). HT29 tumor weight was significantly inhibited by the treatments of LF (1.57 ± 0.19 g) and LA (1.40 ± 0.22 g), when compared with the ones in the control group (*p* < 0.05). What’s more, that in LF + LA group (1.17 ± 0.15 g) was significantly lower than the ones in LF/LA groups (Fig. 5A and 5C).

### Screening of specific metabolites in HT29 cells treated with lactoferrin, linolenic acid and (lactoferrin + linolenic acid) combination

**Figure 6:**
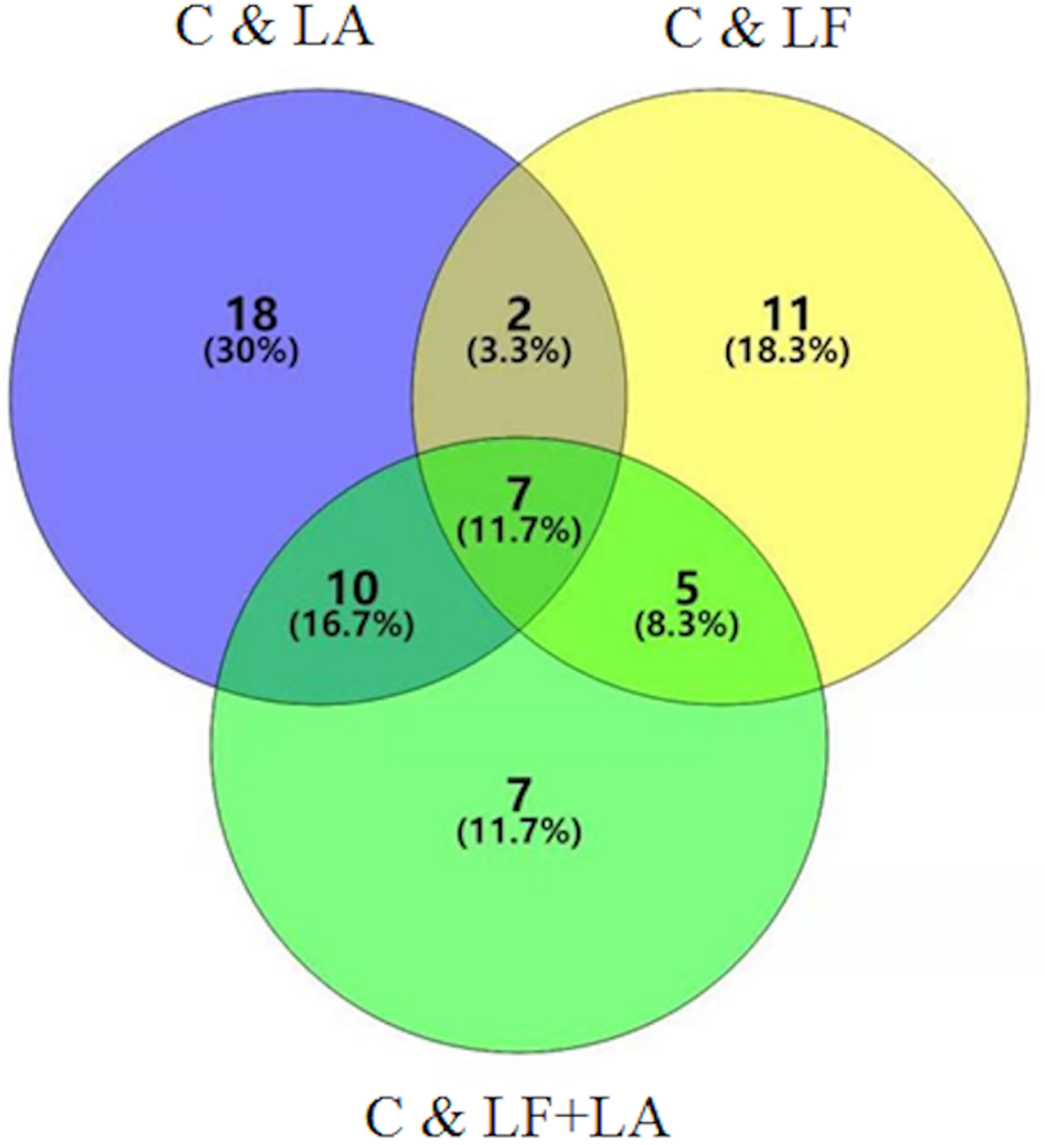
Metabonomics detection in HT29 cells treated with lactoferrin and LA in vitro. Venn diagram shows the overlapping of selected metabolites with changed expressions in control & LF, control & LA, and control & (LF + LA) (*n* = 6).

Combination of data on metabolites from HT29 cells treated with LF, LA, and LF + LA, seven common metabolites were screened out using a VENN diagram (Fig. 6, Table S1).

### Lactoferrin + linolenic acid combination activates AMPK/JNK-related apoptosis pathway

**Figure 7:**
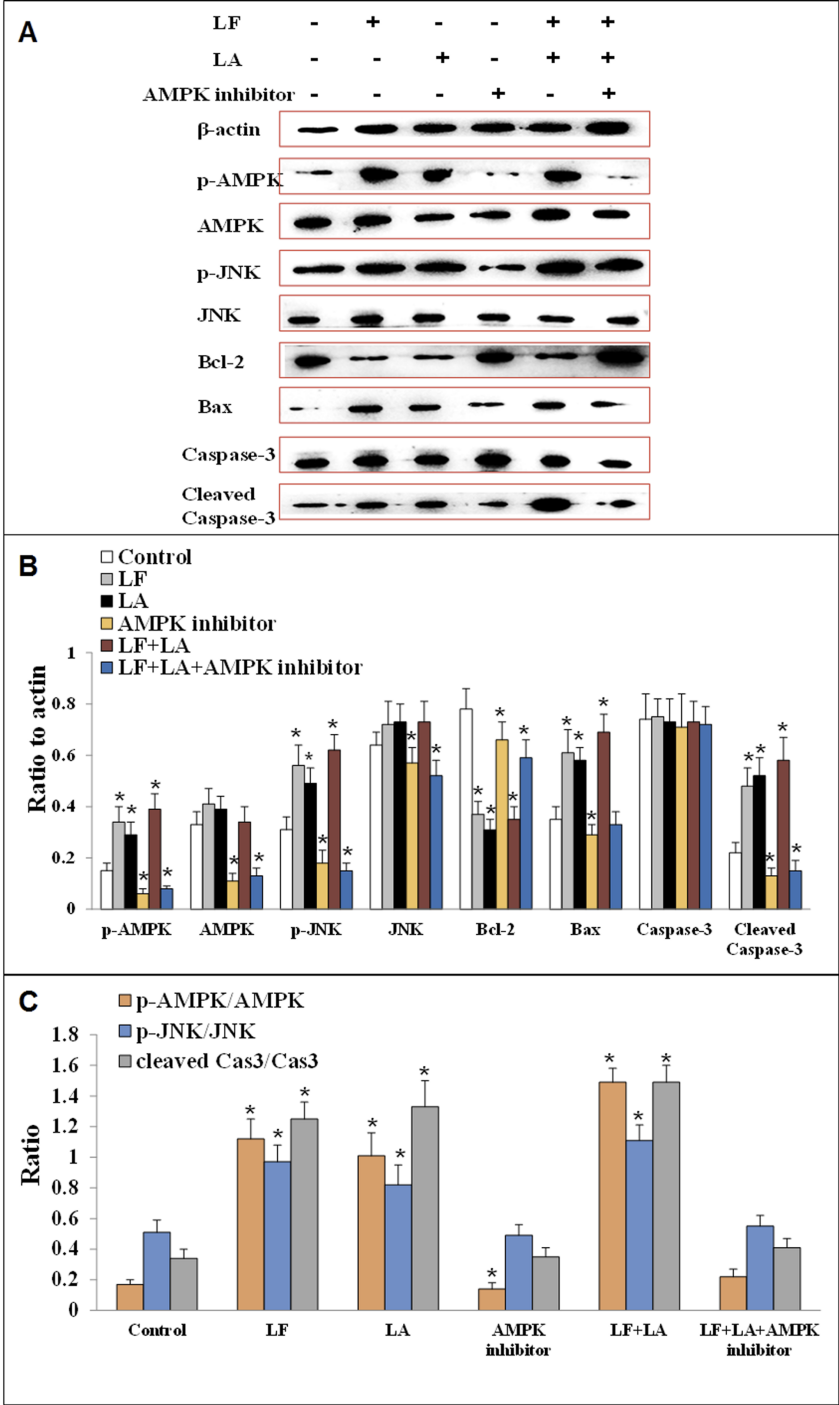
Proteins expression of p-AMPK, AMPK, JNK, p-JNK, Bcl-2, Bax, Caspase-3 and cleaved Caspase-3 in HT29 cells. (A) The protein bands in LF, LA, LF + LA, AMPK inhibitor and LF + LA + AMPK inhibitor treatment groups by western blotting detection. (B) Statistical analysis of the protein bands in (A). (C) The ratio of p-AMPK/AMPK, p-JNK/JNK and cleaved Caspase-3/Caspase-3 in (A). Significant difference from control was observed by variance analysis and *t*-test comparison and all the data were represented as mean ± SD, *p < 0.05, compared with the control, *n* = 3.

To investigate the anti-tumor effect of LF and LA in HT29 tumor models, western blotting was conducted to measure the protein levels of AMPK/JNK-related factors. As shown in Fig. 7, LF, LA, or LF + LA combination upregulated p-AMPK, p-JNK, Bax, and cleaved Caspase-3 significantly (*p* < 0.05) and downregulated Bcl-2 significantly (*p* < 0.05) compared with the control. The levels of p-AMPK, p-JNK, cleaved Caspase-3 and Bax were higher in the LF + LA combination group than in the LF group or LA group. However, the levels of JNK, AMPK and Caspase-3 seemed no obvious change when compared with the control (*p* > 0.05).

To validate the role AMPK in regulating apoptotic factors, HT29 cells were treated with an AMPK inhibitor (dorsomorphin), and the downstream proteins were detected. Results showed that the levels of these proteins (p-AMPK, AMPK, JNK, p-JNK and cleaved Caspase-3) were significantly downregulated, while Bcl-2 was upregulated, compared with the control group (*p* < 0.05). Additionally, those factors in LF + LA + AMPK inhibitor group showed the similar tendencies with the ones in AMPK inhibitor groups, suggesting that LF + LA activate the AMPK/JNK related pathway and ensuing apoptosis factors (Fig. 7).

## Discussion

Colorectal cancer (CRC) usually presents with polyps in the intestinal wall, leading to damage to the large intestine. In early stages, CRC can hardly to be diagnosed and cured. Many patients select chemotherapy rather than surgery for CRC treatment, and 5-fluorouracil (5-FU) is commonly used. In our study, we found that 5-Fu (2.0 μM) could significantly inhibit the HT29 cells viability (about 40%) and induce the apoptosis rate of 37% in HT29 cells, indicating that 5-Fu posed a great threaten for cancer cells growth. However, 5-Fu has serious side-effects of nausea, hair loss, even vomiting. Unlike chemotherapy, natural food ingredients have attracted people’s attention for their inherent biological activities, easy availability, affordability, abilities to modulate a variety of cellular processes, and more importantly, low toxicity in the prevention and treatment of tumors.

The application and addition of LF and unsaturated fatty acids to food products was recently approved. Here, we investigated the effects of LF + OA/DHA/LA combinations on colon cancer and found that LF, OA/DHA/LA, and the combinations induced apoptosis of HT29 cells and inhibited migration and invasion, and the LF + LA combination showed the most potent effect. The in vivo results also demonstrated that the RTV and average tumor weight were rapidly reduced in the LF + LA group compared with control group, indicating the potential of this combination for the intervention of colon cancer. In our previous study, we assessed the effect of LF on HT29 cells both in vitro and in vivo, and found that LF effectively suppressed angiogenesis in HT29 tumor by regulating the VEGFR2/VEGFA/PI3K/Akt/Erk1/2 pathway (Li et al., 2017). Jiang & Lonnerdal (2016) showed that LF could selectively induce the apoptosis in HT29 cells but not in normal human intestinal epithelial cells. Several unsaturated fatty acids also showed antineoplastic effects, especially in colorectal cancer. Llor et al. (2003) investigated the roles of OA and LA in apoptosis, proliferation, and differentiation of Caco-2 and HT29 cells, and demonstrated that the two fatty acids inhibited HT29 cells. An α-lactalbumin-OA (α-LA-OA) complex was constructed and its anti-tumor effect was examined; the results demonstrated that the complex possessed selective tumoricidal activity in murine models of glioblastoma and bladder cancer (Fang et al., 2018).

The anti-tumor activity of LF or OA/DHA/LA alone have been widely validated; however, a comparison of the anti-tumor effects of these combinations (LF + OA/LF + DHA/LF + LA) has been rarely reported. In addition to confirm the anti-tumor roles of LF, three fatty acids, and the combinations, we demonstrated that a possible stronger effect of LF and LA combination on inhibiting HT29 tumors compared with single LF or single LA treatment. Studies have shown a higher expression of LF receptors on tumor cell membranes, which may facilitate LF + PUFAs to blind tumor cells and their subsequent interaction with cell membranes (Wei et al., 2012; Bi et al., 1994; Garré et al., 1992). In addition, fatty acids with unsaturated C18 alkyl chains, such as OA and LA, have been reported to be popular enhancers in the delivery of drugs (Kogan & Garti, 2006). LF + PUFAs induced tumor cell apoptosis (Fig. 2). Among all groups, LF + LA treatment led to a higher level of phosphatidylserine outwards displacement, which is one of the typical characteristics of apoptosis (Arur et al., 2003; Hallgren et al., 2006).

AMPK is a key factor in the regulation of energy metabolism and can be activated by several stimuli, such as cell stress, exercise, and many types of hormones and substances (Dziewulska, Dobrzyn & Dobrzyn, 2010). Recent years, it also has been found to participate in apoptosis of cancer cells through multiple signal pathways. Ben Sahra, Tanti & Bost (2010) showed that the apoptosis of prostate cancer cells was dependent on the AMPK/mTOR pathway. Hwang et al. (2007) observed that AMPK played an important role in mediating apoptosis in HT29 cells. AMPK could transduce multiple extracellular signals similar to JNK, and the activation and phosphorylation of JNK contributed to the induction of tumor cell apoptosis under adverse environmental conditions by activating the expression of pro-apoptotic proteins (such as Bax and executor Caspase-3) and inhibiting the anti-apoptotic protein Bcl-2 (Li et al., 2015). To elucidate the underlying apoptosis molecular mechanism induced by LF + LA, we measured the protein levels of AMPK/JNK-related factors by western blotting. The results demonstrated that the levels of p-AMPK, p-JNK, Bax, and cleaved Caspase-3 increased significantly, whereas the level of Bcl-2 decreased significantly in the LF group, the LA group, and the LF + LA group. Treatment with an AMPK inhibitor suppressed the effect of LF and LA on activating the apoptosis pathway. This suggested that AMPK was the upstream regulator of these apoptotic proteins, and that the effects of LF and LA were mediated by the upregulation of AMPK and JNK. Additionally, the increased expression level of cleaved Caspase-3 was always related with the early apoptosis of tumor cells (Tarhouni-Jabberi et al., 2017), which was also validated in the present study.

## Conclusions

Altogether, the combination of LF + LA exerted strongest anti-tumor effects on HT29 cells by activating the AMPK/JNK pathway and inducing apoptosis. The additive effect between LF and LA needs to be investigated further in the future, which might provide a novel approach to the use of other natural ingredients.

## Supplemental Information

10.7717/peerj.11072/supp-1 Supplemental Information 1 Seven special metabolites screened by metabonomics detection.

10.7717/peerj.11072/supp-2 Supplemental Information 2 Metabonomics raw data.

10.7717/peerj.11072/supp-3 Supplemental Information 3 Chemical information.

10.7717/peerj.11072/supp-4 Supplemental Information 4 Raw data of Western blot in Figure 7.

10.7717/peerj.11072/supp-5 Supplemental Information 5 Figure raw data.

10.7717/peerj.11072/supp-6 Supplemental Information 6 Protocol of Metabonomics detection of HT29 cells.

10.7717/peerj.11072/supp-7 Supplemental Information 7 HT29 cells metabonomics data.

## Additional Information and Declarations

### Data Availability

The following information was supplied regarding data availability: Raw data are available in the Supplemental Files.
