# Supplementary material for: The combination of lactoferrin and linolenic acid inhibits colorectal tumor growth through activating AMPK/JNK-related apoptosis pathway
Source: PeerJ. 2021 May 31;9:e11072. doi: 10.7717/peerj.11072 (PMC8174148; doi:10.7717/peerj.11072)
Supplement: Supplemental Information 1 [file peerj-09-11072-s001.docx]

|  | Description | Formula |
| --- | --- | --- |
| 1 | Hydroxyhexamide | C_15_H_22_N_2_O_4_S |
| 2 | Nummularine B | C_32_H_41_N_5_O_6_ |
| 3 | Penicilloic acid | C_16_H_20_N_2_O_5_S |
| 4 | LysoPC(17:0) | C_25_H_52_NO_7_P |
| 5 | 2-acetyl-1-alkyl-sn-glycero-3-phosphocholine | C_26_H_54_NO_7_P |
| 6 | (2E)-3-phenyl-1-[2,3,4,6-tetrahydroxy-5-(3-methylbut-2-en-1-yl)phenyl]prop-2-en-1-one | C_20_H_20_O_5_ |
| 7 | 1-(2-Furanylmethyl)-1H-pyrrole | C_9_H_9_NO |

**Supplementary Table 1. Seven selected metabolites in HT29 cells**
