# Supplementary material for: The combination of lactoferrin and linolenic acid inhibits colorectal tumor growth through activating AMPK/JNK-related apoptosis pathway
Source: PeerJ. 2021 May 31;9:e11072. doi: 10.7717/peerj.11072 (PMC8174148; doi:10.7717/peerj.11072)
Supplement: Supplemental Information 3 [file peerj-09-11072-s003.docx]

Supplementary table 2 Chemical information

| Reagents | Brand | Address |
| --- | --- | --- |
| Roswell Park Memorial Institute 1640 (RPMI-1640) | GIBCO | Waltham, MA, USA |
| Fetal bovine serum (FBS) | GIBCO | Waltham, MA, USA |
| 1% penicillin/streptomycin | GIBCO | Waltham, MA, USA |
| Colorectal cancer cell line (HT29) | Chinese Academy of Science | Shanghai, China |
| Cell counting kit-8 (CCK-8 kit) | Solarbio | Beijing, China |
| Annexin V/FITC staining apoptosis detection kit | Solarbio | Beijing, China |
| Transwell chambers | Corning | Franklin Lakes, NJ, USA |
| Primary antibodies | Santa Cruz Biotechnology | Santa Cruz, CA, USA |
| Secondary antibodies | Santa Cruz Biotechnology | Santa Cruz, CA, USA |
