# Supplementary material for: The combination of lactoferrin and linolenic acid inhibits colorectal tumor growth through activating AMPK/JNK-related apoptosis pathway
Source: PeerJ. 2021 May 31;9:e11072. doi: 10.7717/peerj.11072 (PMC8174148; doi:10.7717/peerj.11072)
Supplement: Supplemental Information 4 [file peerj-09-11072-s004.pptx]

## Slide 1
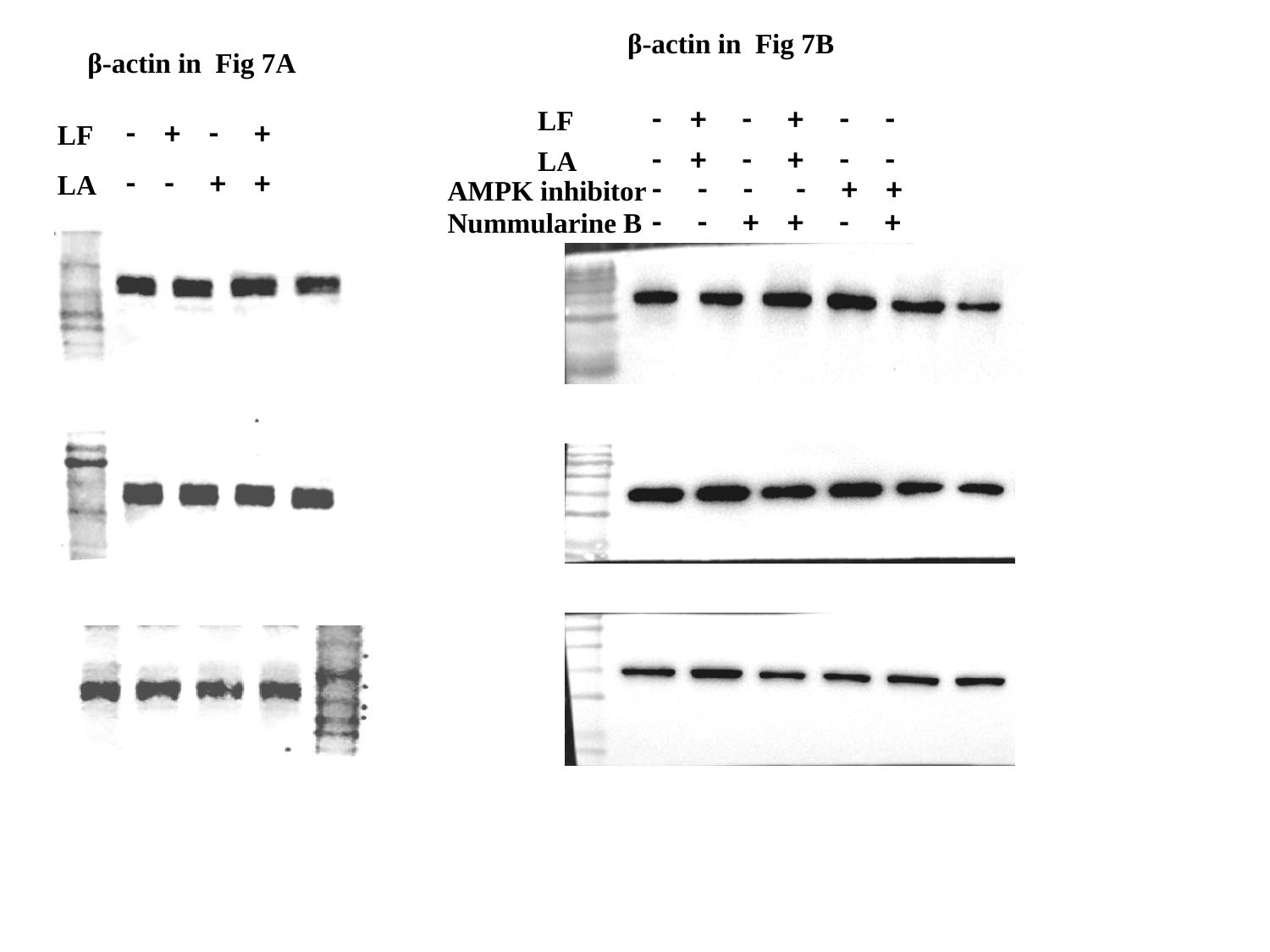

β-actin in Fig 7B
 - + - + - -
LF
 - + - + - -
LA
 - - - - + +
AMPK inhibitor
 - - + + - +
Nummularine B
β-actin in Fig 7A
- + - +
LF
- - + +
LA

## Slide 2
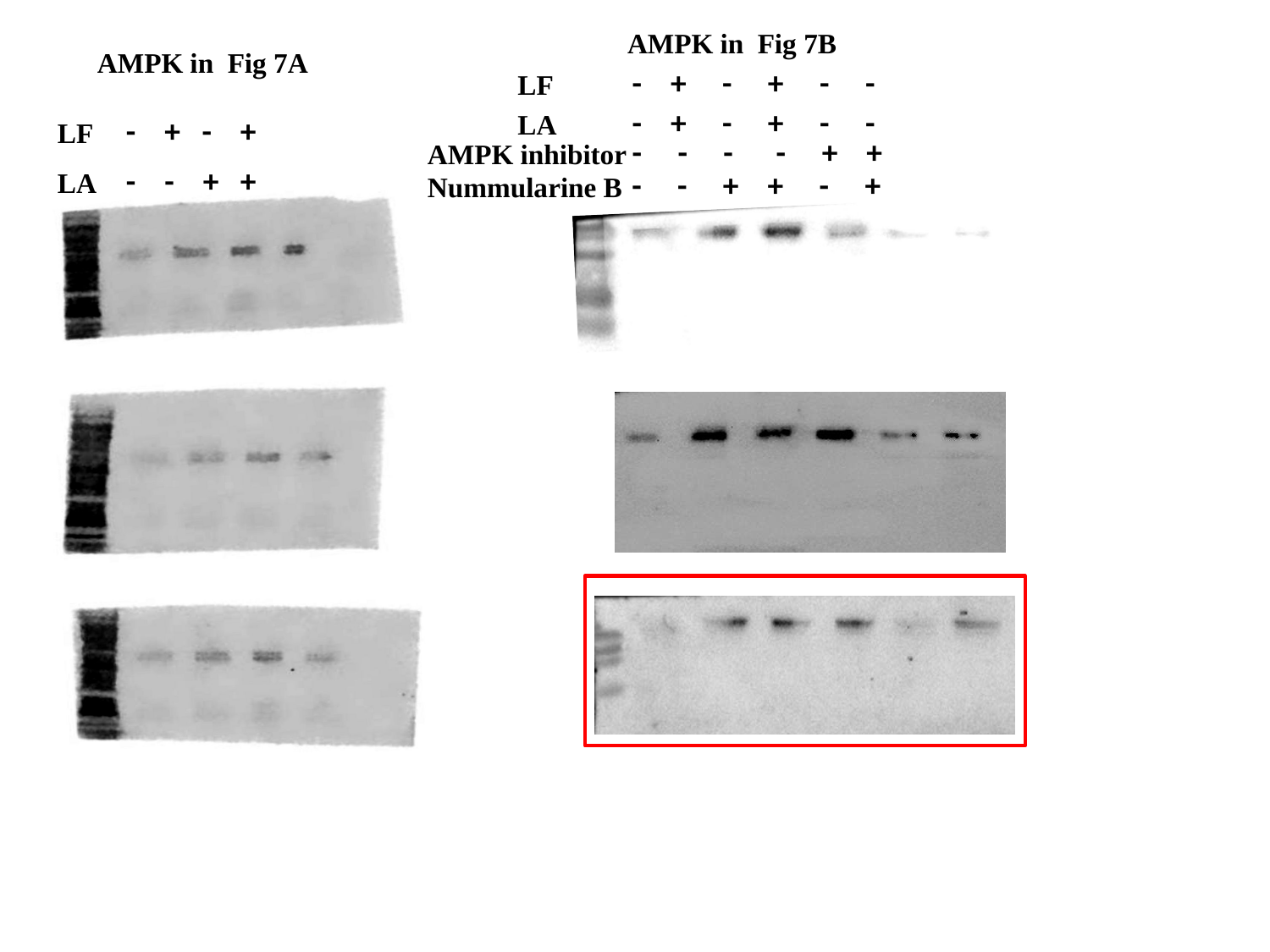

AMPK in Fig 7B
 - + - + - -
LF
 - + - + - -
LA
 - - - - + +
AMPK inhibitor
 - - + + - +
Nummularine B
AMPK in Fig 7A
- + - +
LF
- - + +
LA

## Slide 3
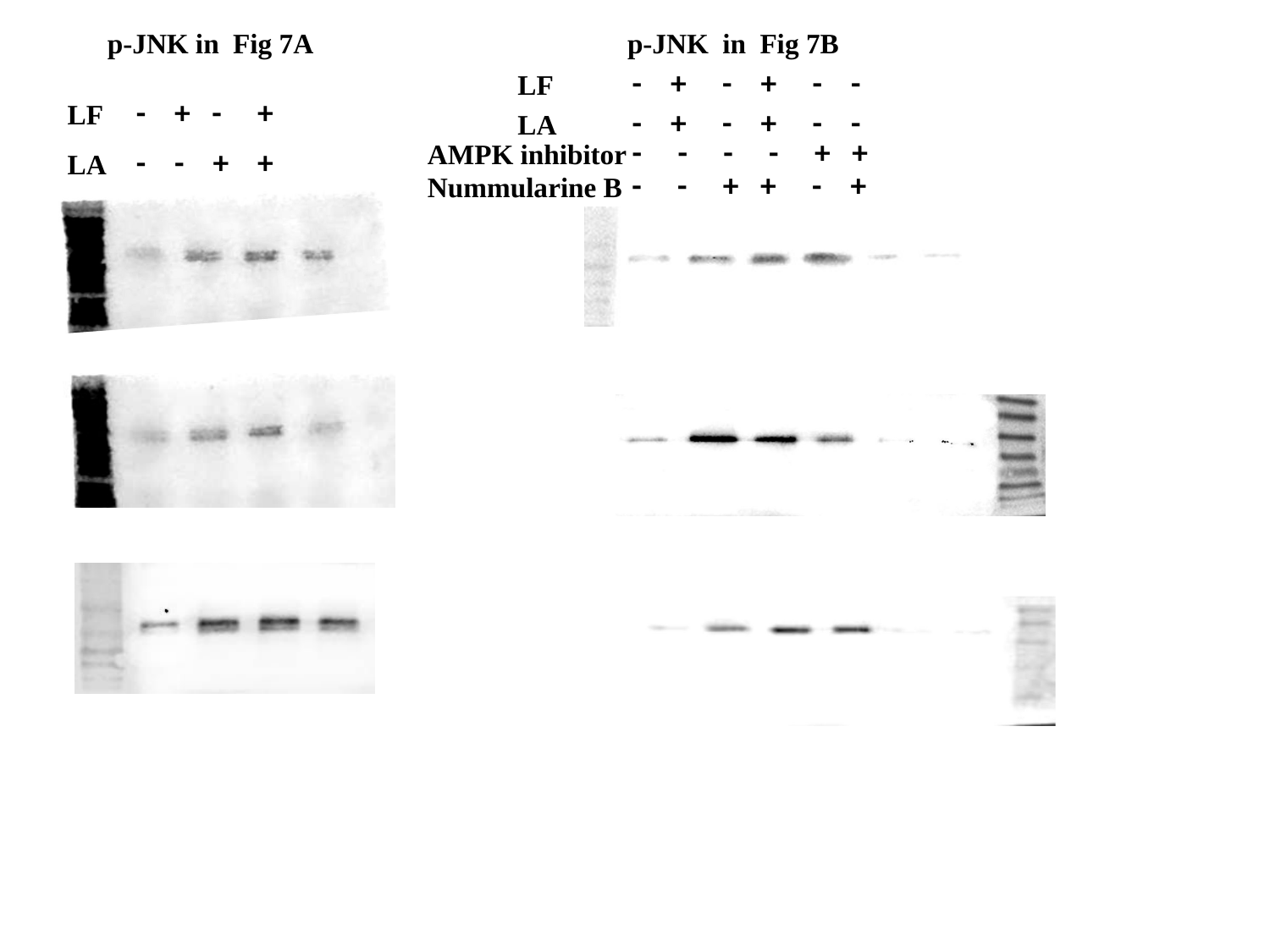

p-JNK in Fig 7A
- + - +
LF
- - + +
LA
p-JNK in Fig 7B
 - + - + - -
LF
 - + - + - -
LA
 - - - - + +
AMPK inhibitor
 - - + + - +
Nummularine B

## Slide 4
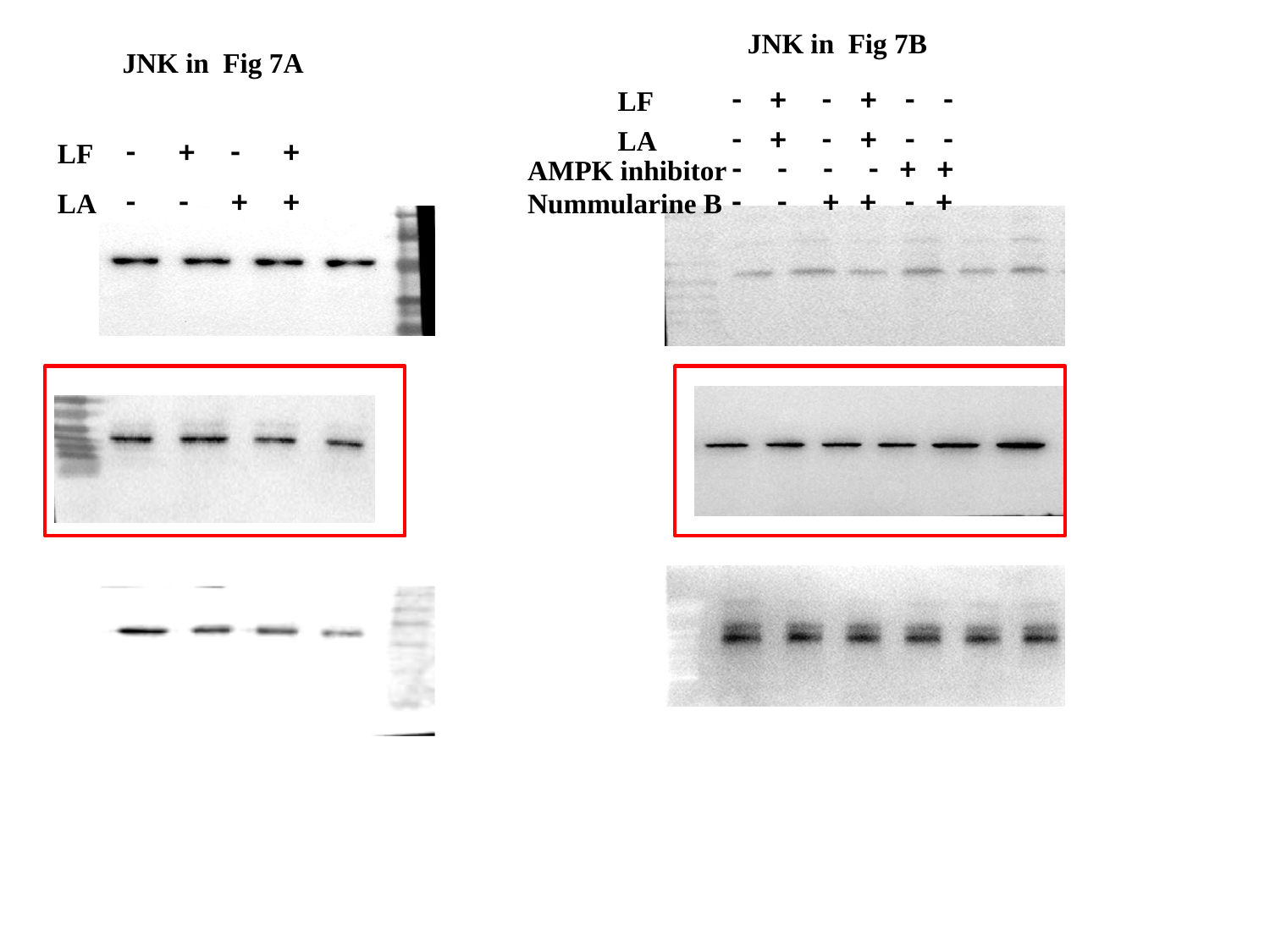

JNK in Fig 7B
 - + - + - -
LF
 - + - + - -
LA
 - - - - + +
AMPK inhibitor
 - - + + - +
Nummularine B
JNK in Fig 7A
- + - +
LF
- - + +
LA

## Slide 5
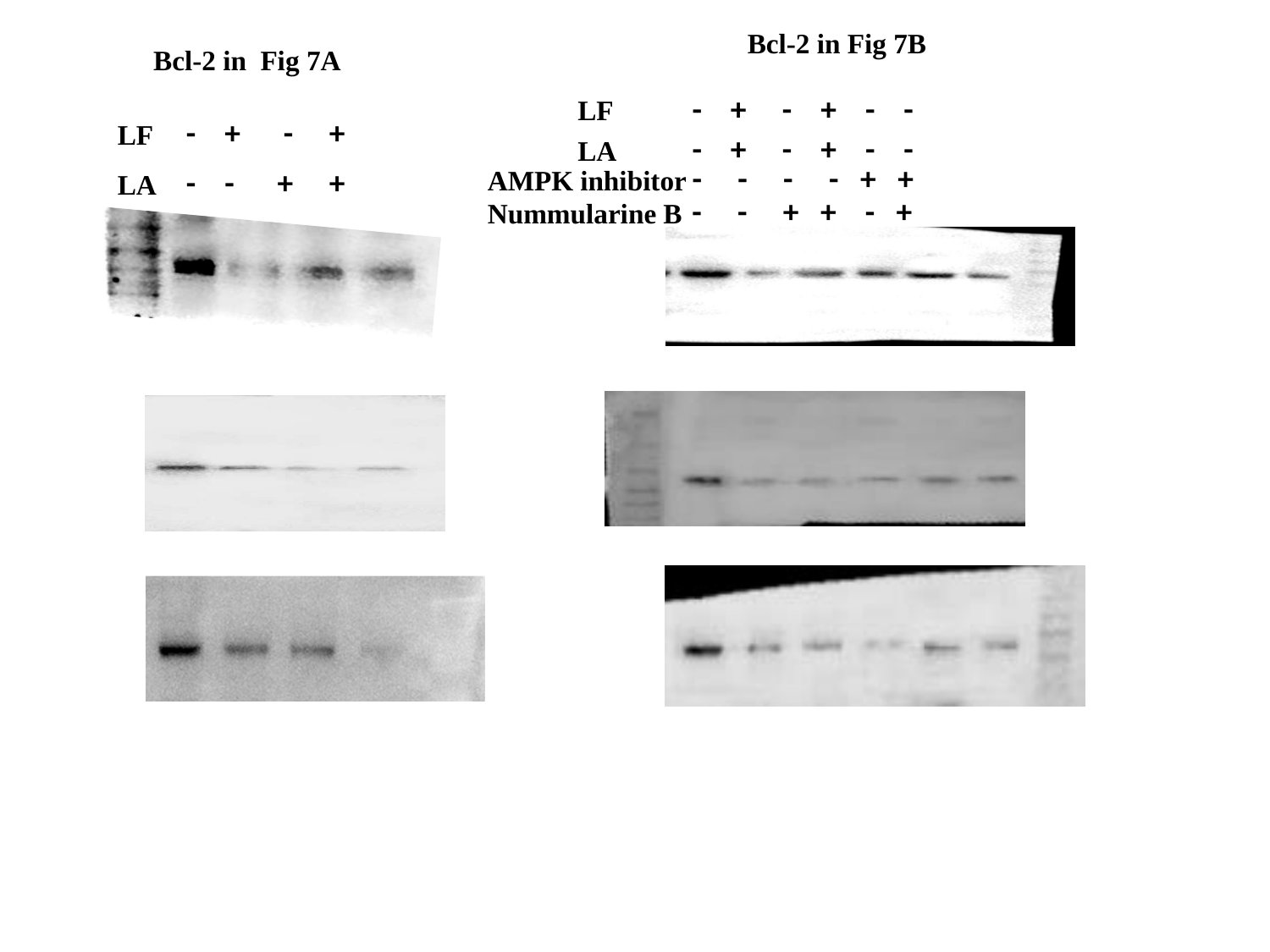

Bcl-2 in Fig 7B
 - + - + - -
LF
 - + - + - -
LA
 - - - - + +
AMPK inhibitor
 - - + + - +
Nummularine B
Bcl-2 in Fig 7A
- + - +
LF
- - + +
LA

## Slide 6
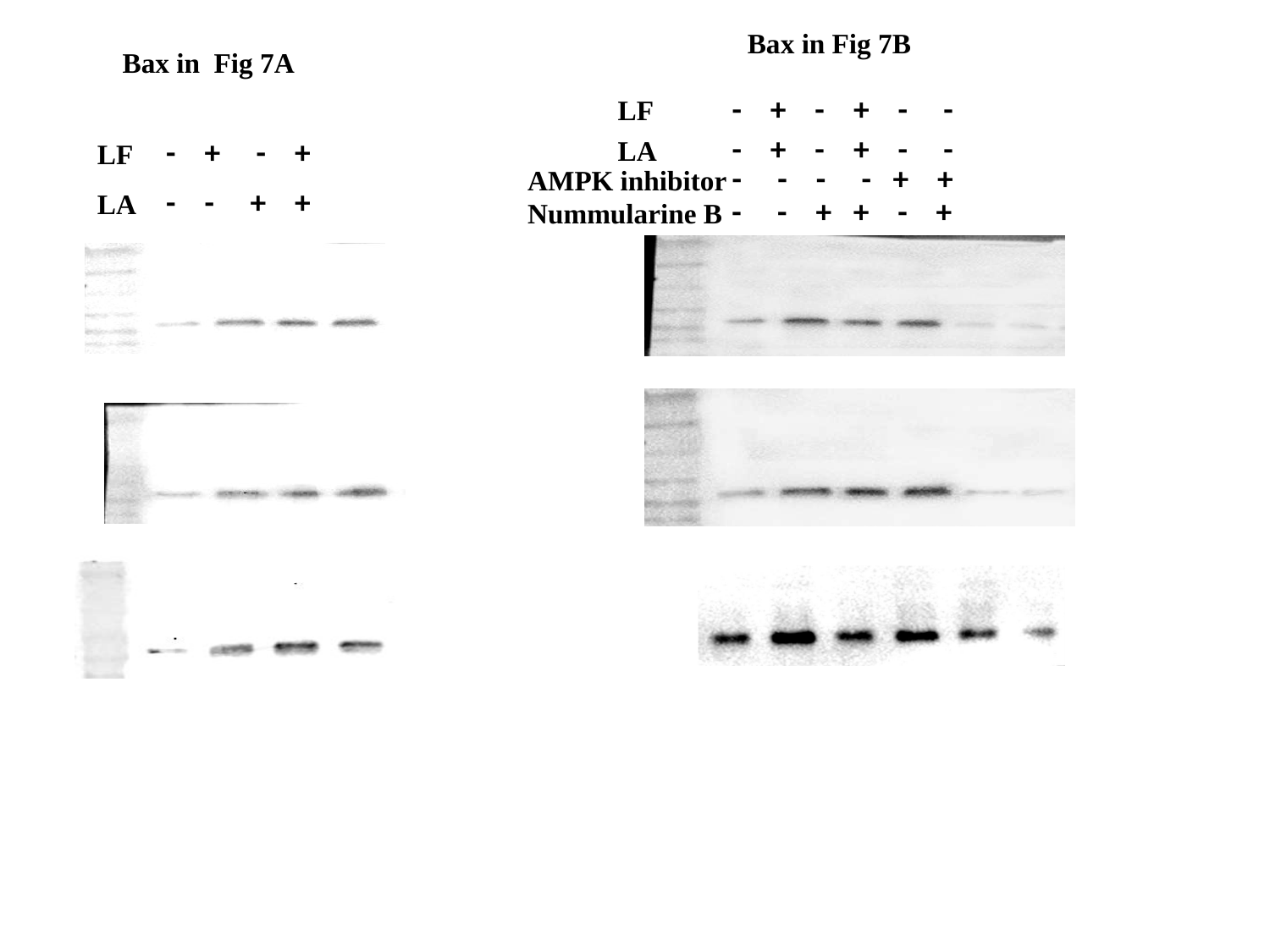

Bax in Fig 7B
 - + - + - -
LF
 - + - + - -
LA
 - - - - + +
AMPK inhibitor
 - - + + - +
Nummularine B
Bax in Fig 7A
- + - +
LF
- - + +
LA

## Slide 7
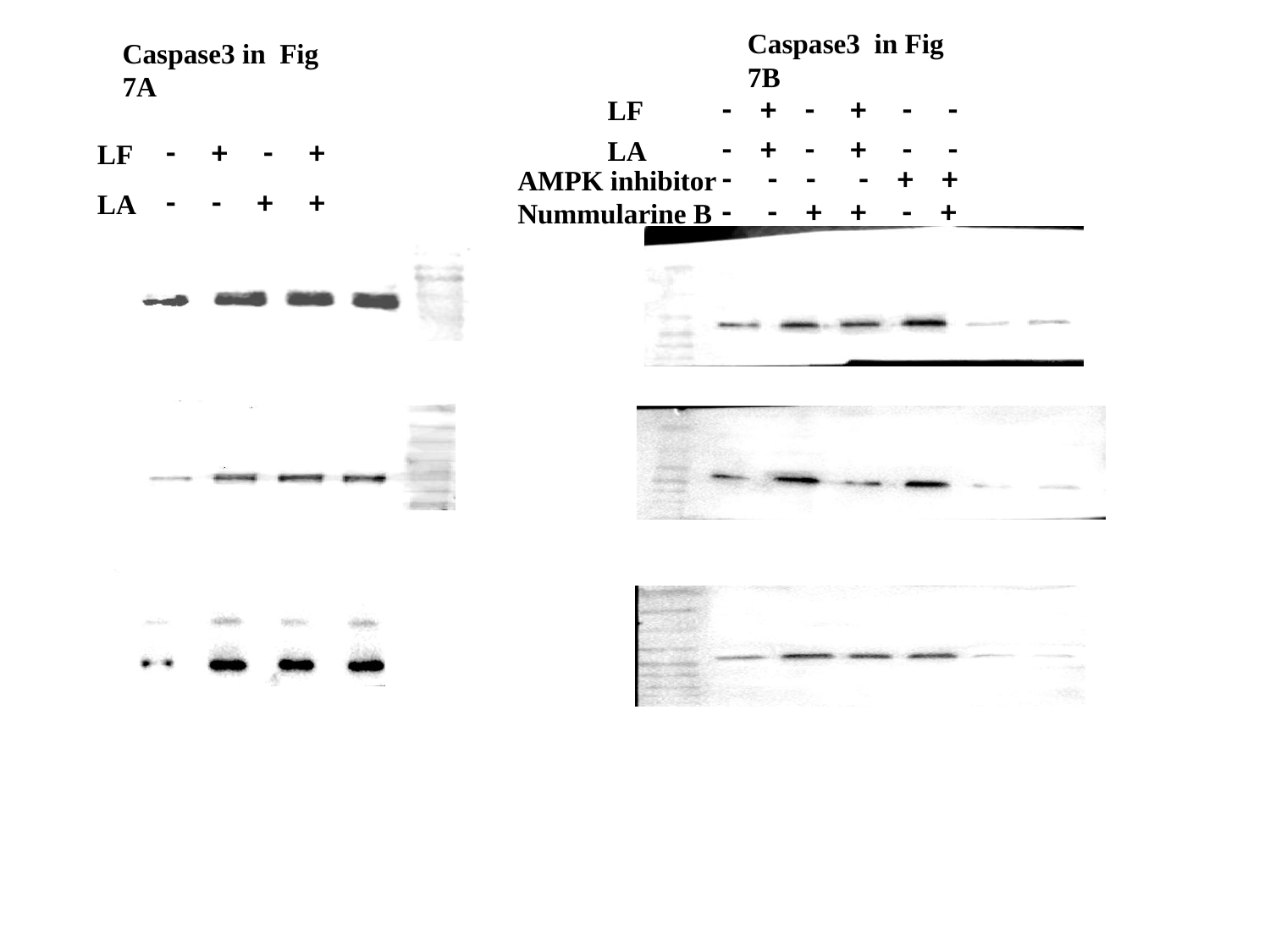

Caspase3 in Fig 7B
 - + - + - -
LF
 - + - + - -
LA
 - - - - + +
AMPK inhibitor
 - - + + - +
Nummularine B
Caspase3 in Fig 7A
- + - +
LF
- - + +
LA
