# Supplementary material for: The combination of lactoferrin and linolenic acid inhibits colorectal tumor growth through activating AMPK/JNK-related apoptosis pathway
Source: PeerJ. 2021 May 31;9:e11072. doi: 10.7717/peerj.11072 (PMC8174148; doi:10.7717/peerj.11072)
Supplement: Supplemental Information 6 [file peerj-09-11072-s006.docx]

**Metabonomics detection of HT29 cells**

Significantly changed metabolites in HT29 cells treated with LF, LA, or (LF + LA) were measured using an ultra-high performance liquid chromatography (UHPLC) system (Dionex Ultimate 3000) equipped with a Waters Column (Acquity BEH C18 1.7 µm, 2.1 × 50 mm) at 40 °C. The mobile phase consisted of water containing 2 mM ammonium formate, and 0.15% formic acid (solvent a, v/v) and acetonitrile containing 0.15% formic acid and 2 mM ammonium formate (solvent b, v/v), with a flow rate of 200 μL/min and the following gradient elution program: 0–1.0 min, 5% b; 1.0–5.0 min, 5% to 60% b; 5.0–8.0 min, 60% to 100% b; 8.0–11.0 min, 100% b; 11.0–14.0 min, 100% to 60% b; 14.0–15.0 min, 60% to 5% b; 15.0–18.0 min, 5% b. The Q-Exactive instrument (Thermo Fisher Scientific) equipped with electrospray ionization in positive and negative switching modes was used to detect the above samples, and the system was calibrated and controlled with X calibur 3.1 and Q-Exactive Tune software. The UHPLC Q-Orbitrap analysis produces large amounts of raw data using TraceFinder software. The data were exported into Excel spreadsheets by Simca-P for the following analyses: PCA (principle components analysis), PLS-DA (partial least squares discriminant analysis), t-test, volcano plot, and VIP (variable importance in projection) plot analysis.
